# Seasonal Influenza Vaccination Policies for Poultry Workers in 33 Pan American Health Organization Member States in 2026: A Review

**DOI:** 10.3390/vaccines14100871

**Published:** 2026-10-01

**Authors:** José Luis Montes, Ximena Juárez, Susana Lloveras, Ana Paula Cordero, Carolina Saenz

**Affiliations:** 1 Medical Affairs Latin America, CSL Seqirus, Olivos C1636, Argentina; ximena.juarez@seqirus.com (X.J.); carolina.saenz@seqirus.com (C.S.); 2 Medical Zoopathology Section, Infectious Diseases Hospital F. J. Muñiz, Uspallata 2272, Buenos Aires C1282AEN, Argentina; sclloveras@gmail.com; 3 Argentine Infectious Diseases Society (SADI), Pasteur 740, Buenos Aires C1028AAH, Argentina; 4 Latin American Society of Travel Medicine (SLAMVI), San Juan, PR 00901, USA; 5 Chair of Infectious Diseases, Faculty of Medical Sciences, University of Buenos Aires (UBA), Paraguay 2155, Buenos Aires C1121ABG, Argentina; 6 Independent Researcher, Olivos C1636, Argentina; acordero@anapaulacordero.com

**Keywords:** influenza, seasonal influenza, influenza vaccine, vaccination policy, vaccination strategies, poultry workers, occupational exposure, One Health, public health

## Abstract

**Background/Objective:** Since 2004, seasonal influenza vaccination has been technically recommended for poultry workers as a public health measure to reduce the risk of co-infection with human and avian influenza viruses and the potential emergence of reassortant strains. The Latin America and the Caribbean (LAC) region has co-circulation of seasonal and avian influenza, with outbreaks in different animal species, including humans. Also, LAC is a major poultry-producing region, with large workforces exposed to domestic poultry, increasing opportunities for virus transmission at the human–animal interface. This review aimed to describe the current state of seasonal influenza vaccination policies across 33 LAC countries and to identify which countries explicitly mention poultry workers. **Methods:** We conducted a literature review with structured and iterative search strategies from the electronic Joint Reporting Form on Immunization (eJRF) and the national health authorities’ websites of the 33 countries. **Results:** As of July 2026, eight countries explicitly included poultry workers among the target groups for seasonal influenza vaccination: Chile, Costa Rica, Cuba, Ecuador, Guatemala, Honduras, Peru and Uruguay. In contrast, 14 countries did not mention poultry workers in their most recent vaccination policies available online. For 11 countries, no publicly available data was identified. **Conclusions:** Substantial gaps exist in the adoption and reporting of seasonal influenza vaccination policies for poultry workers across the LAC region. Most of the region’s major poultry-producing countries do not specifically target this group, nor do most of the countries where outbreaks of avian influenza were recorded. This represents a missed opportunity to strengthen prevention efforts at the human–animal interface.

## 1. Introduction

For over two decades, poultry workers have received the technical recommendation for targeted administration of the seasonal influenza vaccine, as part of a public-health strategy [1]. In 2004, the World Health Organization (WHO), the Food and Agriculture Organization (FAO), and the World Organisation for Animal Health (WOAH, formerly OIE) issued urgent measures in response to the widespread outbreaks of highly pathogenic avian influenza (HPAI). Following a joint technical consultation, they recommended that poultry workers, given their intensive exposure, receive the seasonal influenza vaccine to reduce the risk of coinfection with human and avian influenza viruses, minimizing opportunities for viral reassortment and the emergence of novel influenza viruses with pandemic potential [2,3,4,5].

Genetic reassortment between different influenza A viruses (IAV) could pose a significant public health concern: It could lead to a new IAV with a unique combination of genes from animal and human viruses and adapt to humans in a way that enhances its ability to spread from person to person and intensify a pandemic’s morbidity in the community [6,7,8]. Since avian workers may serve as an interface for the cross-species sharing of influenza viruses, protecting them from human seasonal infection not only reduces its prevalence and severity, but it is also a crucial measure to prevent both the dual infection and the emergence of complicated or adaptive zoonotic infections in humans [9].

Human coinfection with seasonal and avian influenza is a low-frequency but high-impact event and cases can be very severe, with fatal outcomes [10]. In 2024, in a Disease Outbreak News, the WHO confirmed a case of human coinfection with the novel avian influenza A(H10N5) virus and the seasonal influenza A(H3N2) virus activating the notification and response mechanisms established by the International Health Regulations (IHR, 2005) and noting that the patient had not been vaccinated for seasonal influenza [11,12]. The risk of reassortment and the emergence of IAV variants with a higher affinity for human receptors would be even greater if humans were coinfected with seasonal influenza and a preadapted avian virus, such as the one that infected farmers in 2024 in the United States after a spillover from birds to dairy cattle, which then replicated and adapted in other mammals, including human hosts [13]. As a risk mitigation measure, the Centers for Disease Control and Prevention (CDC) recommends seasonal influenza vaccination for rural and poultry workers [14].

The COVID-19 pandemic also reinforced the global adoption of the One Health approach, recognizing that effective preparation for emerging infectious diseases requires coordinated surveillance and response across human, animal, and environmental health sectors [15]. This paradigm was formalized in the Quadripartite One Health Joint Plan of Action (2022–2026), which identifies prevention, early detection, and coordinated management of zoonotic threats as key priorities for pandemic preparedness [16]. Within this framework, integrated influenza surveillance has gained increasing importance through collaboration between the WHO Global Influenza Surveillance and Response System (GISRS) and the WOAH/FAO OFFLU network, whose animal influenza surveillance contributes to risk assessment, the selection of candidate vaccine viruses, and the composition of seasonal and pandemic influenza vaccine [17,18]. Consequently, occupational groups with frequent exposure to infected poultry and other animal reservoirs are increasingly recognized as priority populations within pandemic preparedness strategies.

Latin America is one of the world’s leading poultry producers. Brazil and Mexico are among the top ten poultry-producing nations worldwide, alongside other major contributors to global production. Brazil, Mexico, and Colombia were the three largest broiler producers in 2024, together accounting for most of the regional poultry production; Argentina and Peru were also among the leading ones. The region also includes several of the world’s largest egg-producing industries, which highlights the scale of occupational exposure throughout the poultry production chain [19,20].

Seasonal influenza viruses circulate annually across Latin America and the Caribbean (LAC), where epidemics place a substantial burden on health systems [21]. Meanwhile, since its introduction in the Americas in 2021, the highly pathogenic avian influenza A(H5N1) has expanded geographically in recent years, causing widespread outbreaks in wild birds, backyard flocks, commercial poultry, and mammals in multiple countries across the region, as well as cases in humans [22,23,24]. These concurrent epidemiological scenarios reinforce the importance of protecting occupationally exposed groups such as poultry workers, both to reduce the burden of seasonal influenza and to minimize opportunities for zoonotic transmission and viral reassortment as part of regional and global pandemic preparedness.

In the LAC region, the technical recommendation to identify exposed groups and target them against seasonal influenza vaccination is included in the 2024 regional report of recommendations developed by a group of experts from the recently established Intersectoral Commission for the Prevention and Control of Zoonotic Influenza in the Americas (CIPCIZA) [25,26]. Coordinated by the Pan American Health Organization (PAHO/WHO), the Commission joins human and animal health sectors to prevent and respond to zoonotic influenza threats. The 2024 report was developed to guide national actions and strengthen coordination across sectors to effectively implement the One Health approach.

Despite the co-circulation of avian and seasonal influenza viruses in the LAC region, the strategic importance of its poultry industry, the increasing emphasis on One Health approaches to pandemic preparedness, and the reinforcement of the relevance of seasonal influenza vaccination, especially among occupationally exposed workers [27], data on seasonal influenza vaccination policies for poultry workers in the region remain scarce.

The aim of this study was to review current national influenza vaccination policies across the PAHO Member States in the LAC region, and identify which countries explicitly recommend seasonal influenza vaccination for poultry workers.

## 2. Materials and Methods

This review aimed to describe the current state of seasonal influenza vaccination policies across Latin America and the Caribbean (LAC) countries, with a focus on identifying the presence or absence of the explicit mention of poultry workers, reflecting the WHO and other international technical guidelines on this aspect.

A preliminary, exploratory review was conducted by two of the authors of this study and presented as an abstract and a poster at the ESCMID Global 2025 Congress [28]. That review focused on 19 countries and territories that reported avian influenza outbreaks in birds between 2022 and Epidemiological Week (EW) 39 of 2024. Based on that work, we developed the current 2026 descriptive review, with different inclusion criteria and a wider geographic scope; some countries or territories included in the preliminary assessment are not included in the current analysis of 33.

Additionally, we strengthened the methods with updated and expanded data, to include the WHO/United Nations Children’s Fund (UNICEF) electronic Joint Reporting Form on Immunization (eJRF) database [29], as well as newly released national documents from the participating countries. The analysis was also expanded to incorporate recently published technical guidelines and documents from the WHO, FAO, CDC, WOAH, and a broader assessment of the One Health approach and the post-COVID-19 pandemic context.

### 2.1. Study Design

**Figure 1:**
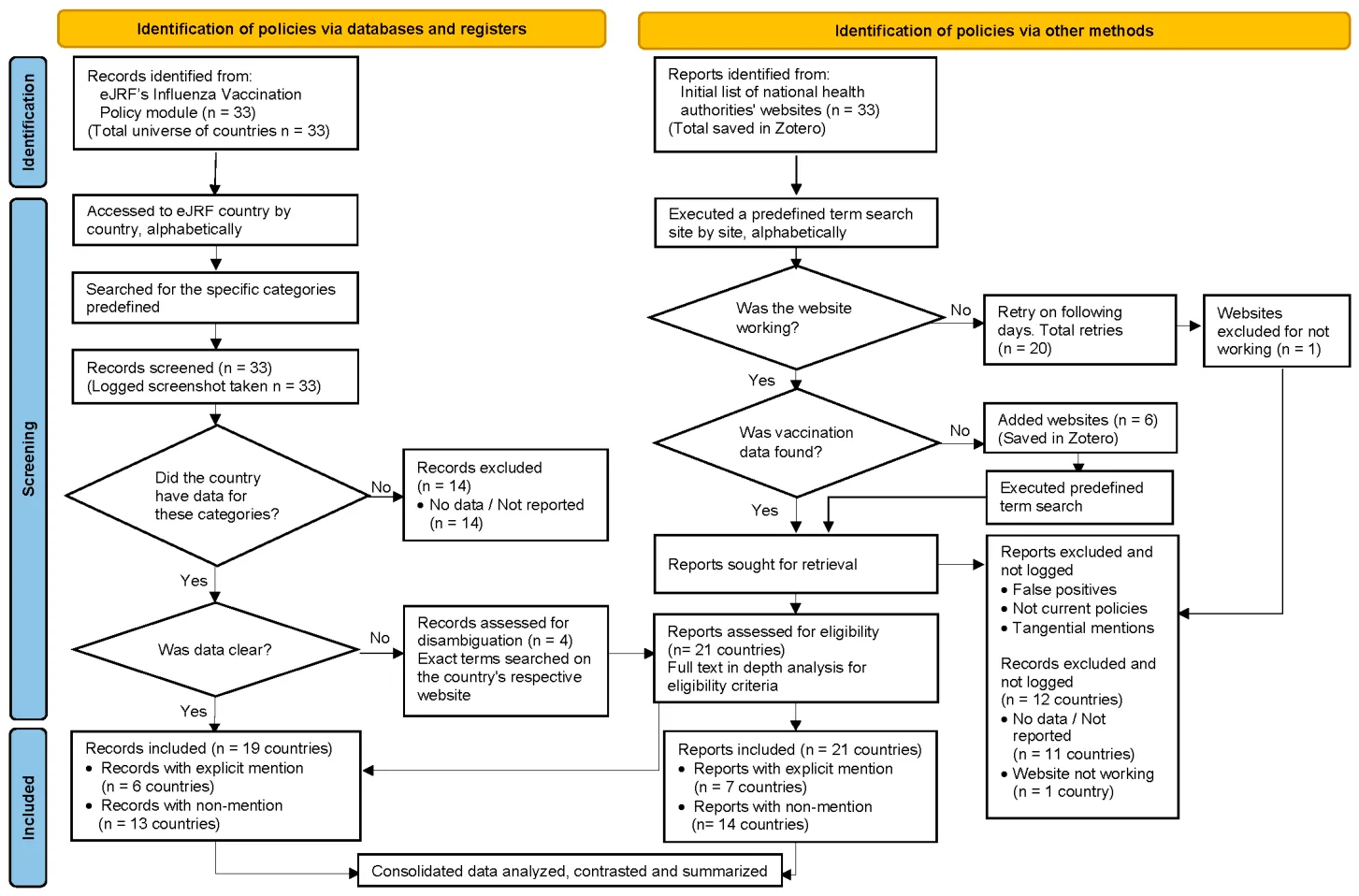
Methodological flowchart of the two-stage search process, adapted from the PRISMA 2020 flow diagram [30] with additional decision points to show the specific process followed in this literature review. The figure shows the identification, screening, and inclusion of records and reports, the main decisions made at each stage, and the procedures used to log the search process and results.

This literature review of national influenza vaccination policies was based on a two-stage process and used structured and iterative search strategies to identify national policies from the eJRF and national sources. It was conducted following a predefined procedure with inclusion/exclusion criteria and decision criteria to identify, screen, and select data. The methodological process is summarized in Figure 1 and described in more detail in the following sections.

### 2.2. Included Countries

A list of countries was defined in advance based on the following selection criteria:

- They must be in the Latin America and Caribbean (LAC) region;
- They must be sovereign states;
- They must be Members States of the Pan American Health Organization (PAHO).

This list remained unchanged throughout the data extraction and verification process.

### 2.3. Data Sources

#### 2.3.1. eJRF WHO/UNICEF Source

Initial information was obtained from the Influenza Vaccination Policy module of the eJRF, accessed via the interactive online browser of the electronical platform [31]. Annually, countries self-report their vaccination data to the JRF. The eJRF is available since 2021.

#### 2.3.2. National Sources

Additional official information was extracted from national health authorities’ websites, as shown in Table 1, and could include any type of document, such as national immunization schedules, influenza national vaccination guidelines, technical guidelines or recommendations, official resolutions, and regulatory documents.

**Table 1:** National sources.

Country	National Health Authorities	Official Website Link
Antigua and Barbuda	Ministry of Health, Wellness and Environment	URL: https://health.gov.ag/ (accessed on 18 July 2026) [32].
Argentina	Ministry of Health	URL: https://www.argentina.gob.ar/salud/ (accessed on 18 July 2026) [33].
Bahamas (The)	Ministry of Health and Wellness	URL: https://www.mohw.gov.bs/ (accessed on 18 July 2026) [34].
Barbados	Ministry of Health and Wellness	URL: https://www.health.gov.bb/ (accessed on 18 July 2026) [35].
Belize	Ministry of Health and Wellness Government of Belize Press Office	URL: https://www.health.gov.bz/ (accessed on 18 July 2026) [36]. URL: https://www.pressoffice.gov.bz/ (accessed on 18 July 2026) [37].
Bolivia	Ministry of Health and Sports	URL: http://www.minsalud.gob.bo (accessed on 18 July 2026) [38].
Brazil	Ministry of Health	URL: https://www.gov.br/saude/pt-br (accessed on 18 July 2026) [39].
Chile	Ministry of Health Public Health Institute	URL: https://www.minsal.cl/ (accessed on 18 July 2026) [40]. URL: https://Ispch.gob.cl (accessed on 19 July 2026) [41].
Colombia	Ministry of Health and Social Protection Expanded Program on Immunization	URL: https://www.minsalud.gov.co/ (accessed on 18 July 2026) [42]. URL: https://vacunacion.minsalud.gov.co/ (accessed on 18 July 2026) [43].
Costa Rica	Ministry of Health	URL: https://www.ministeriodesalud.go.cr/ (accessed on 18 July 2026) [44].
Cuba	Ministry of Public Health	URL: https://salud.msp.gob.cu/ (accessed on 18 July 2026) [45].
Dominica	Ministry of Health, Wellness and Social Services	URL: https://dominica.gov.dm/ministries/health-wellness-and-social-services (accessed on 18 July 2026) [46].
Dominican Republic	Ministry of Public Health	URL: https://www.msp.gob.do/ (accessed on 19 July 2026) [47].
Ecuador	Ministry of Public Health	URL: https://www.salud.gob.ec (accessed on 19 July 2026) [48].
El Salvador	Ministry of Health	URL: https://www.salud.gob.sv/ (accessed on 19 July 2026) [49].
Grenada	Ministry of Health, Wellness & Religious Affairs	URL: https://www.gov.gd/health (accessed on 19 July 2026) [50].
Guatemala	Ministry of Public Health and Social Assistance Guatemalan Social Security Institute	URL: https://www.mspas.gob.gt/ (accessed on 19 July 2026) [51]. URL: https://www.igssgt.org/ (accessed on 19 July 2026) [52].
Guyana	Ministry of Health	URL: https://health.gov.gy/ (accessed on 19 July 2026) [53].
Haiti	Ministry of Public Health and Population	URL: https://www.mspp.gouv.ht/ (accessed on 19 July 2026) [54].
Honduras	Health Department	URL: https://www.salud.gob.hn/ (accessed on 19 July 2026) [55].
Jamaica	Ministry of Health and Wellness	URL: https://www.moh.gov.jm/ (accessed on 19 July 2026) [56].
México	Health Department	URL: https://www.gob.mx/salud (accessed on 19 July 2026) [57].
Nicaragua	Ministry of Health	URL: https://www.minsa.gob.ni/ (accessed on 19 July 2026) [58].
Panama	Ministry of Health	URL: https://minsa.gob.pa/ (accessed on 19 July 2026) [59].
Paraguay	Ministry of Public Health and Social Welfare Expanded Program on Immunization	URL: https://www.mspbs.gov.py/ (accessed on 19 July 2026) [60]. URL: https://pai.mspbs.gov.py/ (accessed on 19 July 2026) [61].
Peru	Ministry of Health	URL: https://www.gob.pe/minsa (accessed on 19 July 2026) [62].
Saint Kitts and Nevis	Ministry of Health and Social Security	URL: https://www.gov.kn/health/ (accessed on 20 July 2026) [63].
Saint Lucia	Ministry of Health, Wellness and Nutrition	URL: https://health.govt.lc/ (accessed on 20 July 2026) [64].
Saint Vincent and Grenadines	Ministry of Health, Wellness, Environmental Health and Energy	URL: https://health.gov.vc/health/ (accessed on 20 July 2026) [65].
Suriname	Ministry of Health, Welfare and Labour Bureau for Public Health (BOG)	URL: https://gov.sr/ministeries/ministerie-van-volksgezondheid-welzijn-arbeid/ (accessed on 20 July 2026) [66]. URL: https://bogsuriname.com/ (accessed on 21 July 2026) [67].
Trinidad y Tobago	Ministry of Health	URL: https://health.gov.tt/ (accessed on 20 July 2026) [68].
Uruguay	Ministry of Public Health	URL: https://www.gub.uy/ministerio-salud-publica/home (accessed on 20 July 2026) [69].
Venezuela	Ministry of Popular Power for Health	URL: https://mpps.gob.ve/ (accessed on 20 July 2026) [70].

### 2.4. Search Strategy

The review was conducted on a country-by-country basis in two stages:

#### 2.4.1. Stage 1: Structured Search from the Influenza Vaccination Policy Module of the WHO/UNICEF eJRF

We used the browser’s filters to access data corresponding to each country for the period between 2020 and 2025. For each country, variables related to influenza vaccination recommendations were extracted, with particular attention focused on the following categories:

- “Is influenza vaccination recommended for other groups?”;
- “Additional information on the recommendation for other groups”.

The data were recorded exactly as presented on the platform, without filling in missing values or modifying the original entries.

#### 2.4.2. Stage 2: Structured and Iterative Search Process of National Sources

For each country, the official health-authorities’ websites were consulted.

Considering the diversity of languages across LAC countries, and to minimize the risk of conducting a more detailed search in some countries than in others, the search was conducted using a predefined list of terms and keywords to identify influenza vaccination policies with explicit mentions of poultry workers with a combination of translated and conceptually equivalent terms across four official languages: Spanish, Portuguese, French and English. Table A1 presents this list of concepts and terms, specifying the language in which each country’s search was conducted.

The review was conducted with no restrictions regarding the type of document. Documents were eligible for inclusion if they provided specific information on the national seasonal influenza policy, regardless of whether poultry workers were explicitly mentioned, as shown in Figure 1. If there were more than one version of influenza vaccination policies, we considered the most recent one.

#### 2.4.3. Analysis of Ambiguous Categories

In cases where the eJRF included the phrases “other prioritized groups” or equivalent, a specific search was conducted in the national sources to identify which population groups were covered by that term in each country.

This process involved reviewing the exact text reported and other variables to determine whether there were additional descriptions that could clarify the scope of the recommendation.

### 2.5. Eligibility Criteria

A predefined inclusion and exclusion criteria was applied to ensure the inclusion of relevant documents, as shown in Table 2.

### 2.6. Data Collection and Analysis

Data extracted from the WHO/UNICEF eJRF was collected in a document available upon request that included dated screenshots, their respective URLs, as well the related URL from the national sources when a disambiguation was needed. For the collection and management of evidence from the national health authorities’ websites the following predefined strategies were implemented considering the iteration process of the search and the instability of national public websites: (1) Between 18 and 20 July 2026 the home page of each website was captured and saved using Zotero software (version 9.0.6, 64-bit), with each URLs indexed to preserve metadata. (2) When the main authority website did not provide vaccination data, an alternative and more specific one was search, captured and saved in Zotero in the same or following days. (3) Only the documents that passed the predefined screen process and underwent filters (for relevance, false positives, policy recentness, direct mention), were saved in Zotero.

Two reviewers independently screened records and reports and extracted relevant information. Any discrepancies were resolved through discussion and consensus. When agreement could not be reached, a third reviewer was consulted. Final inclusion decisions and data classifications were established collaboratively by all the authors.

After we identified the countries that explicitly mentioned poultry workers in their seasonal influenza vaccination policies, we summarized the results in tables by country, source, and type of mention using three categorized criteria:

- Explicit mention;
- Non-mention;
- No publicly available data.

Then, we conducted a narrative, descriptive analysis.

## 3. Results

Seasonal influenza vaccination policies from 33 PAHO Member States in LAC were reviewed where available data. The findings are presented below by country according to the available official sources, relevant content, and the level of inclusion of poultry workers in national seasonal influenza vaccination programs, as shown in Table 3.

The findings were also classified by country and by three categorized criteria: explicit mention of poultry workers; non-mention; and no publicly available data, as shown in Table 4.

### 3.1. Countries with Seasonal Influenza Vaccination Policies for Poultry Workers

Of the 33 PAHO Member States in LAC, the minority (*n* = 8, 24.24%) explicitly include poultry workers among the target groups for seasonal influenza vaccination in their policies, reflecting WHO technical guidelines on this aspect: Chile, Costa Rica, Cuba, Ecuador, Guatemala, Honduras, Peru and Uruguay.

Costa Rica included poultry workers among the priority groups for receiving the seasonal influenza vaccine in 2026, according to information on its Ministry of Health’s website; however, the eJRF does not yet reflect this change, as the information is reported annually by each country.

Another case that demonstrated the usefulness of the two-stage review methodology, with analysis of ambiguous categories, was that of Guatemala. The finding of an explicit mention in its 2025 vaccination schedule was only possible after we conducted an exact text search, but not on its Ministry of Health’s website, but on the Guatemalan Social Security Institute website. This search followed the detection of an ambiguous phrase identified during the data extraction from the eJRF: “Other groups prioritized by risk” (“Otros grupos priorizados por riesgo” in the original Spanish language).

In the cases of Ecuador and Peru, explicit mentions were not found in the eJRF or their respective vaccination schedules published on the websites of their ministries of health [81,82]. Rather, they were found in specific documents identified through more detailed searches. In Ecuador, the mention was found in the 2026 Immunization Manual for Health [75]. In Peru, it was found in a technical health regulation establishing the 2026 national immunization schedule, under the section on at-risk groups and for poultry workers exposed to infected live or dead birds [78].

In Chile, our searches of the two data sources led to recent and coincidental findings.

The explicit mentions of poultry workers in the influenza vaccination policies from Cuba, Honduras, and Uruguay could only be found in data on the eJRF from 2021 and 2023 and then verified on the Cuban and Uruguayan health authority websites. The website of the Honduran Department of Health was not available, and the explicit mention was found in a technical guidance on another source, the Virtual Health Library of Honduras [83].

### 3.2. Countries with No Mention of Poultry Workers in Their Seasonal Influenza Vaccination Policies

**Figure 2:**
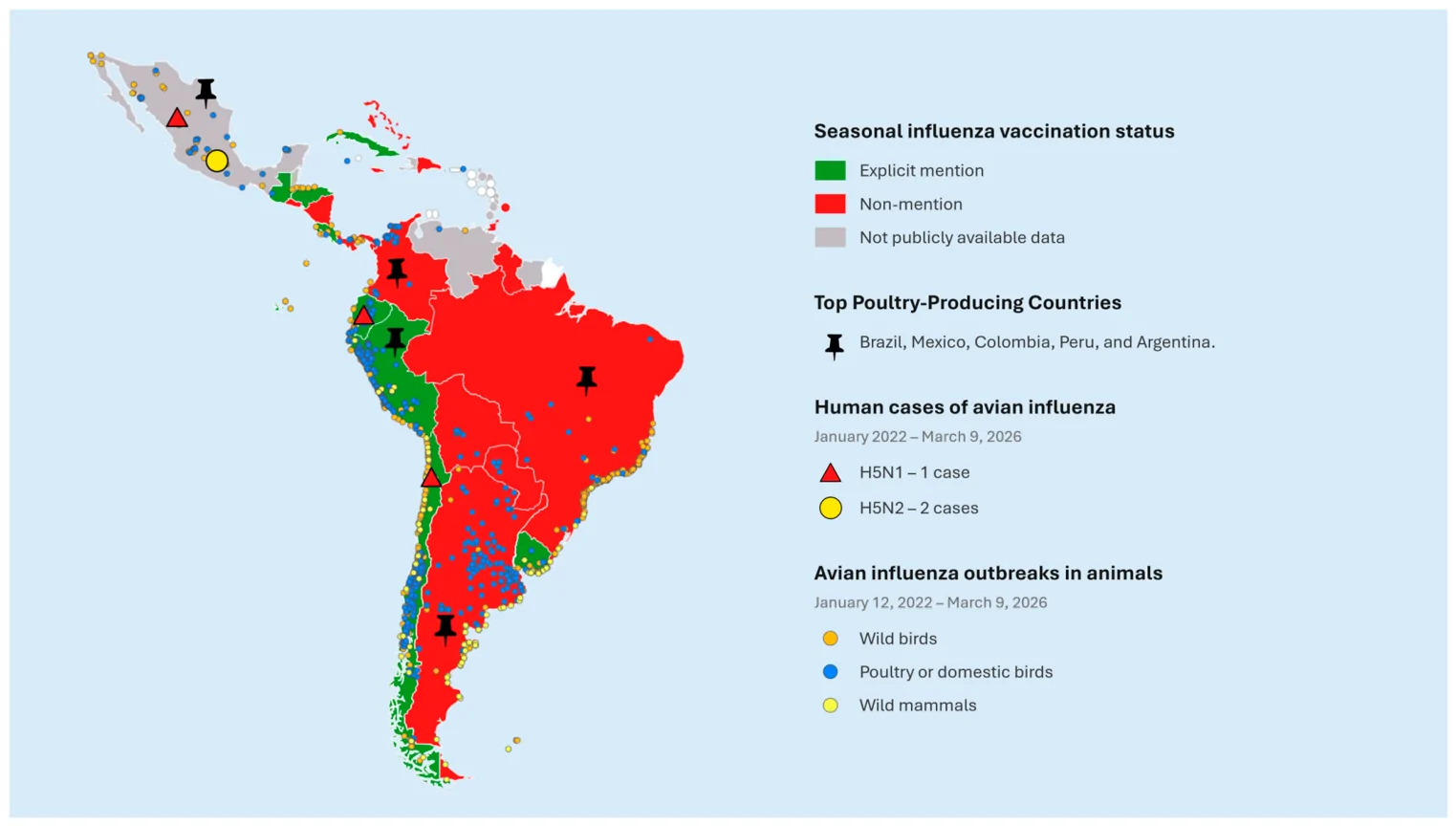
33 Latin American and the Caribbean PAHO Member States map combining three levels of data: 1. Results of this review: explicit mention of poultry workers in seasonal vaccination policies; non-mention; and not publicly available data. 2. Avian influenza outbreaks since 2022 [22,97]. 3. Main poultry producers countries.

The greatest percentage of countries (*n* = 14, 42.42%) did not mention poultry workers in their most recent seasonal influenza vaccination policies. These countries were: Argentina, Bahamas, Barbados, Bolivia, Brazil, Colombia, Dominican Republic, El Salvador, Guyana, Jamaica, Nicaragua, Panama, Paraguay, Trinidad and Tobago, as shown in Table 4 and in Figure 2.

There is a special case with Panama as it included poultry workers in its 2020 report to the eJRF and in an article on its Ministry of Health official website in 2021 [84], but the 2026 seasonal influenza technical guidelines did not include them.

### 3.3. Countries with No Publicly Available Data

For one-third of the countries (*n* = 11, 33.3%), no information on influenza vaccination policies for other groups was found either in the eJRF or on the websites of their health authorities. Those countries were: Antigua and Barbuda, Belize, Dominica, Grenada, Haiti, Mexico, Saint Kitts and Nevis, Saint Lucia, Saint Vincent and the Grenadines, Suriname, and Venezuela, as shown in Table 4 and Figure 2. Though, in previous years, Belize, Dominica, and El Salvador reported in that category to the eJRF that seasonal influenza vaccination was available for the general population (Table 3).

## 4. Discussion

### 4.1. Key Findings in a Scenario of Co-Circulation of HPAI and Seasonal Influenza Viruses

Despite longstanding international recommendations promoting seasonal influenza vaccination among poultry workers to mitigate the risk of co-infection with human and avian influenza viruses and the potential emergence of novel reassorting strains, our review identified that only eight of the 33 PAHO Member States in the LAC region (Chile, Costa Rica, Cuba, Ecuador, Guatemala, Honduras, Peru and Uruguay) explicitly include this population as a target group in their vaccination policies. In 14 countries, no mention of poultry workers was found in the official documents available online.

According to the information publicly available online identified during the study period, 42.42% (14/33) of the countries included in this review did not reflect the WHO recommendation on this aspect, although the region is experiencing avian influenza outbreaks since its introduction in North America in 2021. Between epidemiological week (EW) 2 of 2022 and EW 9 of 2026, 5744 outbreaks of HPAI A(H5N1) have been notified cumulatively in 21 countries of the Americas Region, in diverse bird and mammalian species. Also, 75 human infections caused by HPAI A(H5N1), including two deaths, were reported in five countries (United States of America, Mexico, Canada, Chile, and Ecuador), between 20 April 2022 and 9 March 2026 [22], as shown in Figure 2. From the three LAC countries with human cases, only two, Chile and Ecuador, have in place a vaccination policy for poultry workers.

In the first half of 2026, outbreaks of HPAI in animals were recorded in eight LAC countries: Argentina, Brazil, Chile, Colombia, Honduras, Mexico, Peru, and Uruguay [85]. Among them, only four (Chile, Honduras, Peru and Uruguay) have seasonal influenza vaccination policies for poultry workers. Vaccinating poultry workers against the seasonal influenza viruses is considered a public health measure [86], as some of the human influenza pandemics (in 1957, 1968, and 2009) were caused by the reassortment of genes from human and avian viruses [87]; cases of human coinfection, while rare, are cause for concern, as the recent one from 2024 [11].

### 4.2. Technical, Reporting and Communicational Challenges

Another significant finding was the considerable heterogeneity in the availability, accessibility, and clarity of official information regarding seasonal influenza vaccination policies for the general population and for poultry workers across the region. Information retrieval was frequently challenging because recommendations were dispersed across different sources, inconsistently reported, or not publicly available: in 11 countries, no supporting evidence could be identified despite exhaustive searches on eJRF databases and on their national authorities’ websites. And even in countries such as Cuba, Costa Rica, Ecuador, and Peru—where explicit mentions were found in certain documents—these were not included in their vaccination schedules available online on their health authorities’ websites.

These findings highlight important challenges: technical and communicational gaps; breaches in the self-report information or underreporting from each country to the eJRF; and the need to standardize the “Other risk group” category, and inside it, the vaccination policies for occupationally exposed groups.

Similar challenges were noted in a recent worldwide review of seasonal influenza vaccination policies in the JRF with a different scope [88]. While a 2019 report on influenza in Latin America, with a narrower geographical focus and a different methodological approach, showed discrepancies with our findings regarding the inclusion of poultry workers, it only identified three countries (Chile, Panama, and Paraguay) [89]; however, the sources are no longer available. In contrast, our review revealed that, although Panama’s 2020–2021 national vaccination recommendations explicitly identified poultry workers as a target group for seasonal influenza vaccination, this recommendation was omitted from subsequent immunization schedules. In Paraguay, poultry workers were not mentioned in either the eJRF or the health authorities’ websites.

### 4.3. Relevant Findings Due to the Strategic Importance of the Latin American (LATAM) Region in the Global Poultry Industry

LATAM is among the world’s leading producers of poultry meat and eggs, with countries such as Brazil, Mexico, Colombia, Peru and Argentina accounting for a substantial proportion of global production, as shown in Figure 2. Though reliable and comparable workforce estimates are unavailable across countries due to the lack of standardized reporting and the substantial contribution of informal employment, this workforce is routinely exposed to domestic poultry, increasing opportunities for bidirectional transmission of avian influenza viruses at the human–animal interface. Despite the economic importance of this sector and the number of workers with potential occupational exposure, only one of these major producer countries—Peru—explicitly includes poultry workers among the target groups for seasonal influenza vaccination in its publicly available national immunization program, and only in cases of exposure to infected birds. The Colombian 2026 National Technical and Operational Guideline for Seasonal Influenza Vaccination provides a mechanism to extend influenza vaccination to additional at-risk populations, subject to risk assessment and authorization by the Ministry of Health and Social Protection; but neither in that category are poultry workers currently included among them [90].

If we consider the complete list of poultry producer LATAM countries, we identified that only seven (Peru, Ecuador, Chile, Guatemala, Honduras, Costa Rica, and Uruguay) included poultry workers in its seasonal influenza vaccination policies, from the total of 17 ranked in 2025 as follows: Brazil, Mexico, Colombia, Peru, Argentina, Venezuela, Ecuador, Bolivia, Chile, the Dominican Republic, Guatemala, Honduras, Panama, Costa Rica, Paraguay, El Salvador, and Uruguay [91].

Worldwide, some of the greater avian producer countries, such as the United States [14], Russia [92], Thailand [93], France [94] and the United Kingdom [95,96], have implemented seasonal influenza vaccination policies for poultry workers.

### 4.4. Other Implications for Influenza Pandemic Preparedness

Strengthening seasonal influenza vaccination programs may contribute to broader pandemic preparedness capacities. Previous analyses of influenza vaccination in the Americas have highlighted that countries with established seasonal influenza immunization programs were better positioned to rapidly implement and expand COVID-19 vaccination strategies compared with countries where adult influenza vaccination platforms were weak or absent. These experiences suggest that seasonal influenza vaccination programs represent not only a disease-specific intervention but also an essential component of sustainable immunization infrastructure [98]. Studies also show that having seasonal influenza vaccination programs in place can contribute to ensuring vaccine acceptance during a pandemic [99].

In this context, improving access to and uptake of seasonal influenza vaccination among occupational groups at higher risk of exposure, such as poultry workers, may strengthen vaccine confidence and acceptance of other adult vaccines targeting priority populations at increased risk of severe disease and complications [98]. During the 2024 H5N1 outbreak, the CDC expanded its response providing free seasonal influenza vaccination for poultry, dairy, and livestock workers, together with outreach and educational initiatives [100]. Similarly, the United Kingdom recommends seasonal influenza vaccination for workers occupationally exposed to birds. However, recent evidence indicates that vaccine uptake remains suboptimal, emphasizing that formal recommendations alone are insufficient and should be accompanied by sustainable funding, accessible vaccination services, and targeted communication strategies [95].

The LAC region has much to do in order to prepare and protect poultry workers, as we revealed that only eight (24.24%) of the 33 PAHO Member States have seasonal vaccination recommendations in place for this highly exposed group, some with no easy to access information, and only one of them among the regional main poultry producers; and that 11 countries (33.3%) have no available information for risk groups.

### 4.5. Study Limitations

Despite its strengths, this review has limitations in different areas that should be considered when interpreting the findings. The lack of publicly available information for some countries, differences in institutional transparency, variability in accessibility, completeness, and updating of official documents, as well as technological barriers such as not working or underperforming websites may have prevented the retrieval of existing policies. Although we conducted a multilanguage search, language barriers should be considered as well. Another key limitation is the dynamic nature of national policies, regulations, and legislation. The scope of this review was limited to describing the explicit mention of poultry workers in national seasonal influenza policies available online and did not assess policy effectiveness, implementation processes, or barriers and facilitators to implementation.

## 5. Conclusions

This literature review identified substantial gaps both in the adoption and reporting of seasonal influenza vaccination policies for poultry workers across the LAC region, where there have been multiple outbreaks of HPAI viruses in different animal species, including human cases, and co-circulation with seasonal influenza viruses. In the context of the recent establishment of the PAHO Intersectoral Commission for the Prevention and Control of Zoonotic Influenza in the Americas (CIPCIZA) and its ongoing development of recommendations to effectively implement the One Health approach, these findings may provide an evidence basis for future research and policy improvements to enhance national and regional pandemic influenza readiness.

As the Commission also highlights the importance of identifying the groups with the highest occupational risk, these findings are particularly relevant. The review found that, despite the region’s strategic role in the global poultry industry, most of its largest poultry-producing countries do not currently include poultry workers among the target groups for seasonal influenza vaccination. This represents a missed opportunity to strengthen health protection in populations with high exposure to avian influenza viruses.

Also, having robust seasonal influenza vaccination programs can support immunization infrastructure, foster public trust in vaccination, and enhance preparedness for future public health emergencies [99]. Expanding seasonal influenza vaccination policies for poultry workers needs to be taken into consideration within a comprehensive One Health framework that integrates human, animal, and environmental health. Greater regional harmonization of recommendations and improved accessibility of official immunization policies would represent important steps toward strengthening pandemic preparedness in a region with a critical role in the global poultry industry.

## Figures and Tables

**Table 2:** Predefined inclusion and exclusion criteria for report selection.

Criterion	Inclusion Criteria	Exclusion Criteria	Rationale/Justification
Geographical and Jurisdictional Scope	Reports issued by national authorities of the selected countries.	Reports from countries outside the selected sample; or issued by provincial, municipal or local authorities.	To focus on national oriented reports.
Web Availability	Reports publicly available online on official websites.	Reports not publicly available online on official websites.	To reflect online availability.
Information availability	Reports with sufficient information.	Reports with no information or insufficient information.	To assess policy content.
Thematic Scope and Content	Reports addressing national seasonal influenza vaccination policies, programs, guidelines, schedules that explicitly state target groups.	Reports unrelated to seasonal influenza national vaccination policies; or lacking the explicit state of target groups.	To collect available documents on seasonal influenza vaccination policies to document if they explicitly mentioned poultry workers or not.
Document Type and Institutional Authorship	Any official report (web publication/announcement, press release, guideline, document) issued by national or subnational health ministries, public health agencies, or equivalent governmental authorities.	Reports (web publications and documents) lacking national official endorsing.	To include policies, regardless of their format, with authoritative endorsement.
Temporality	The most recent policies within the study period.	Outdated policies within the study period.	To analyze the most current available version of each policy.
Language	Reports in Spanish, Portuguese, French, and English languages.	Reports in other than the selected languages.	To minimize language bias.

**Table 3:** Poultry workers mention in influenza vaccination policies LAC countries in 2026.

Country		WHO/UNICEF eJRF		National Health Authorities’ Websites
	Explicit Mention ^1^ (2020–2025 Period)	Ambiguous Mention ^2^	Other	Explicit Mention ^3^ (January–July 2026 Period)
Antigua and Barbuda	No	No	ND	ND
Argentina	No	No	No	No
Bahamas	No	No	“General population”	No
Barbados	No	No	No	No
Belize	No	No	ND “Persons outside the target groups”	ND
Bolivia	No	No	No	No
Brazil	No	No	No	No
Chile	Yes (2025/2020)	No	No	Yes [71,72]
Colombia	No	No	No	No
Costa Rica	No	No	No	Yes [73]
Cuba	Yes (2021/2020)	No	No	Yes [74]
Dominica	No	No	ND “Any person who wants vaccine”	ND
Dominican Republic	No	Yes	No	No
Ecuador	No	No	No	Yes [75]
El Salvador	No	No	“Otras edades de demanda”	No
Grenada	No	No	ND	ND
Guatemala	No	Yes	No	Yes [76]
Guyana	No	No	No	No
Haiti	No	No	ND	ND
Honduras	Yes (2021)	No	No	ND ^4^
Jamaica	No	No	No	No
Mexico	No	No	ND	ND
Nicaragua	No	No	No	No
Panama	Yes (2020)	No	No	No [77]
Paraguay	No	Yes	No	No
Peru	No	No	ND	Yes [78]
Saint Kitts and Nevis	No	No	ND	ND
Saint Lucia	No	No	ND	ND
Saint Vincent and Grenadines	No	No	ND	ND
Suriname	No	No	ND	ND
Trinidad y Tobago	No	No	ND	No [79]
Uruguay	Yes (2023)	No	No	Yes [80]
Venezuela	No	No	ND	ND

Abbreviations: eJRF, Influenza Vaccination Policy module of the WHO/UNICEF electronic Joint Reporting Form on Immunization; ND, No Data. ^1^ The following phrases were found in the eJRF and categorized as explicit mentions: “Granjas avícolas” (for Chile, year 2025); “Trabajadores de granjas avícolas” (for Cuba, year 2021); “Trabajadores de granjas avícolas” (for Honduras, year 2021); “Granjas avícolas” (for Panama, year 2020); “Personal con exposición a aves” (for Uruguay, year 2023). ^2^ The following phrases were found in the eJRF and categorized as ambiguous and led to specific searches in the national sources: “Personas que por su trabajo se ven expuestas” (for Dominican Republic); and “Otros grupos priorizados por riesgo” (for Guatemala). ^3^ The following phrases were found on national health authorities’ websites and categorized as explicit mentions: “Trabajadores avícolas” (for Chile); “Personas que trabajan en granjas avícolas” (for Costa Rica); “Trabajadores avícolas” (for Cuba); “Trabajadores de avícolas” (for Ecuador); “Trabajadores de ganadería: avicultura” (for Guatemala); “Trabajadores avícolas” (for Peru); and “Trabajadores en granjas avícolas” (for Uruguay). ^4^ The official website of the Honduran Health Department could not be accessed during the study period because it was down or returning corrupted output.

**Table 4:** Seasonal influenza vaccination policy for poultry workers in PAHO LAC countries.

Inclusion of Poultry Workers	Number of Countries (*n* = 33) (%)	Countries
Explicit mention ^1^	8 (24.24)	Chile, Costa Rica, Cuba, Ecuador, Guatemala, Honduras, Peru and Uruguay.
Non-mention	14 (42.42)	Argentina, Bahamas, Barbados, Bolivia, Brazil, Colombia, Dominican Republic, El Salvador, Guyana, Jamaica, Nicaragua, Panama, Paraguay and Trinidad and Tobago.
No publicly available data	11 (33.3)	Antigua and Barbuda, Belize, Dominica, Grenada, Haiti, Mexico, Saint Kitts and Nevis, Saint Lucia, Saint Vincent and Grenadines, Suriname, Venezuela.

^1^ Overall results were classified as “Explicit mention” when it was identified in at least one of the two predefined data sources.

## Data Availability

The original contributions presented in this study are included in the article. Further inquiries can be directed to the corresponding author.

## Abbreviations

The following abbreviations are used in this manuscript:

CIPCIZA	Intersectoral Commission for the Prevention and Control of Zoonotic Influenza in the Americas
CDC	Centers for Disease Control and Prevention
eJRF	Electronic Joint Reporting Form on Immunization
EW	Epidemiological Week
FAO	Food and Agriculture Organization
GISRS	Global Influenza Surveillance and Response System
HPAI	Highly pathogenic avian influenza
IAV	Influenza A viruses
IHR	International Health Regulations
JRF	Joint Reporting Form on Immunization
LAC	Latin America and the Caribbean
PAHO	Pan American Health Organization
UNICEF	United Nations Children’s Fund
WHO	World Health Organization
WOAH	World Organisation for Animal Health

## Appendix A

**Table A1:** Search terms by concept and language in national sources.

Concepts	Database: National Health Authorities’ Websites Search Terms Used ^1^			
	Search Terms in Spanish ^2^	Search Terms in Portuguese ^3^	Search Terms in French ^4^	Search Terms in English ^5^
Seasonal influenza vaccination	“Influenza”; “Gripe”; “Gripe estacional”; “Vacunación”; “Inmunización”.	“Gripe”; “Vacinação”; “Vacina”; “Imunização”.	“Grippe”; “Vaccination”.	“Influenza”; “Flu”; “Seasonal influenza”; “Immunization”; “Vaccine”; “Vaccination”.
Vaccination schedule/Vaccination technical guidelines	“Calendario de vacunación”; “Lineamientos técnicos de vacunación”.	“Imunização”; “Diretrizes técnicas”.	“Calendrier de vaccination”.	“Vaccine schedule”; “Vaccination schedule”; “Technical guidelines”.
Poultry workers/Avian workers	“Trabajadores avícolas”; “Granjas”; “Granjas de aves”; “Aviar”; “Avícola”.	“Trabalhadores avícolas”; “Fazenda”; “Fazendas de aves” “Aves”; “Avicultura”.	“Travailleurs avicoles”; “Fermes avicoles”; “Avicole”.	“Poultry workers”; “Poultry farms”; “Poultry”; “Contact with birds”.
Exposed workers	“Trabajadores expuestos”.	“Trabalhadores expostos”.	“Travailleurs exposés”.	“Exposed workers”.
Risk groups	“Grupos priorizados”; “Grupos de riesgo”.	“Grupos priorizados”; “Grupos de risco”.	“Groupes à risque”.	“Risk groups”.

^1^ The identical concept was used in all languages. Equivalent search terms were used depending on the country’s language. ^2^ Search terms for Spanish-language countries: Argentina; Bolivia; Chile; Colombia; Costa Rica; Cuba; Dominican Republic; Ecuador; El Salvador; Guatemala; Honduras; Mexico; Nicaragua; Panama; Paraguay; Peru; Uruguay; and Venezuela. ^3^ Search terms for Portuguese-language country: Brazil. ^4^ Search terms for French-language country: Haiti. ^5^ Search terms for English-language countries: Antigua and Barbuda; Bahamas (The); Barbados; Belize; Dominica; Grenada; Guyana; Jamaica; Saint Kitts and Nevis; Saint Lucia; Saint Vincent and Grenadines; Suriname (its health authority official website is available in English); and Trinidad and Tobago.
